# Enterovirus D68 VP1 and VP3 Determine Neurotropism in Human Spinal Cord Organoids

**DOI:** 10.3390/v18060619

**Published:** 2026-05-28

**Authors:** Jessica E. Packard, Jennifer E. Jones, Gal Yovel, Megan Culler Freeman

**Affiliations:** 1 Department of Pediatrics, University of Pittsburgh School of Medicine, Pittsburgh, PA 15224, USA; jep329@pitt.edu (J.E.P.); jennyjones_xyz@pitt.edu (J.E.J.); gal.yovel@pitt.edu (G.Y.); 2 Institute of Infection, Inflammation, and Immunity in Children (i4Kids), Pittsburgh, PA 15224, USA

**Keywords:** enterovirus, enterovirus D68, acute flaccid myelitis, neurotropism, organoids, recombinant virus

## Abstract

Enterovirus D68 (EV-D68) is a non-polio enterovirus that can cause a polio-like paralysis condition, acute flaccid myelitis (AFM). EV-D68-associated AFM cases waned in the US after 2018, and the reasons for this are unknown. It has recently been demonstrated that EV-D68 containing point mutations in viral structural proteins VP1 and VP3 resulted in decreased paralysis in different neonatal mouse models. However, phenotypes of these mutations in a human multicellular central nervous system (CNS) model are unknown. We hypothesize that mutations in VP1 and VP3 will similarly direct neurotropism in human spinal cord organoids (hSCOs). To investigate this, we recreated viruses with mutations in VP3 (I88V) or VP1 (L1I/N2D/T98A/E283K or L1P/V148A/K282R) and infected hSCOs. We found that VP3 I88V and VP1 L1I/N2D/T98A/E283K resulted in decreased titer and viral protein staining, consistent with attenuated neurovirulence in previously published murine models. We also found through immunofluorescence that VP1 L1P/V148/K282R mutations altered cellular tropism, primarily infecting glial cells rather than neuronal cells. When these mutations were combined, their effects on neurotropism were not additive. Sequence analysis of recently circulating EV-D68 strains reveals that VP3 I88 and VP1 E283 have remained the dominant amino acid residues since 2014, whereas VP1 sites 1, 2, and 98 have higher population diversity, indicating that these residues may be contributing to newly reduced neurovirulence after 2018.

## 1. Introduction

Enterovirus D68 (EV-D68) is a non-enveloped enterovirus within the *Picornaviridae* family originally isolated from patient respiratory samples in 1962 [1]. Today, EV-D68 is considered a reemerging virus due to increased circulation, severe respiratory illness and new neurovirulence. EV-D68 infections are linked to acute flaccid myelitis (AFM), a polio-like illness resulting in paralysis, primarily in children [1,2].

Prior studies investigating EV-D68 determinants of neuropathogenesis have used mouse models to study the relationship between EV-D68 infection and the development of paralysis. Two groups identified mutations in structural proteins VP1 and VP3 as primary drivers of neuropathogenesis [3,4]. Yeh et al. 2020 found that in EV-D68 US/IL/14-18952 (EV-D68 18952), introduction of a mutation in VP3 at site 88 from isoleucine to valine (VP3 I88V), as present in MO/14-18949, resulted in decreased paralysis and viral titer in day seven C57BL/6-derived Tg21 mice with type I interferon receptor knockout (Tg21/IFNR-ko) after intraperitoneal (IP) injection [3]. The authors found that five-day-old C57BL/6-derived Tg21 mice infected with VP3 I88V via intracranial (IC) injection had significantly decreased viral titer in the brain and spinal cord compared to EV-D68 18952. Additional VP1 mutations, L1P, V148A, K282R, and G283E were investigated. They found that VP1 mutations in L1P and K282R/G283E by IP injection resulted in decreased viral titer and paralysis in seven-day-old mice at 24 h post infection (hpi) and 72 hpi.

Leser et al. 2024 also sought to evaluate EV-D68 neuropathogenesis determinants by comparing neurovirulent EV-D68 18952 and avirulent CA/14-4231 (CA4231) [4]. They found that while EV-D68 18952 results in paralysis after intramuscular (IM) injection in Swiss Webster interferon-deficient (mitochondrial antiviral signaling protein (MAVS^−/−^)) mice bred on a pure C57BL/6 background, CA4231 does not. Through systematic evaluation of the differences between the strains, they discovered that four VP1 amino acid mutations present in CA4231 (L1I/N2D/T98A/E283K) were the primary drivers of EV-D68 neurovirulence.

The discordance between the mutations responsible for driving neuropathogenesis may be reconcilable for a few reasons [3,4]. Primary differences between the two studies include the use of mouse genetic backgrounds, inoculation route used (IM vs. IP vs. IC injection), and mapping to different avirulent strains (MO vs. CA). To assess EV-D68 determinants of neurovirulence independent of these variables, we sought to investigate if these same mutations conferred a similar phenotype in a human multicellular model with intact elements of innate immunity [5]. Specifically, we generated recombinant EV-D68 (rEV-D68) strains to match the VP3 mutation from Yeh et al. (rVP3-I88V-MO), three amino acid changes in VP1 described by Yeh et al. (rVP1-3AA-MO), and four amino acid changes described by Leser et al. (rVP1-4AA-CA) [3,4].

Using a previously established multicellular human spinal cord organoid (hSCO) model for EV-D68 neurotropism, we evaluated the replication competence of each rEV-D68. All rEV-D68-containing mutations replicated in our hSCO model to differing degrees. Both rVP3 I88V-MO (strain from Yeh et al. [3]) and rVP1 4AA-CA (strain from Leser et al. [4]) produced significantly decreased viral titer in hSCOs at 48 and 96 hpi when compared to the reference strain, consistent with phenotypic conclusions from the murine models. These findings suggest that while these viruses may have potential defects in accessing the CNS from the initial infection site in murine models, they also have decreased replication capacity in CNS cells. Additionally, infection of hSCOs with rEV-D68 18952, rVP3-I88V-MO, and rVP1-4AA-CA resulted in the colocalization of the viral protein with neurons, whereas rVP1-3AA-MO primarily colocalized with glial cells, suggesting that these three amino acid changes in VP1 altered cellular tropism. Because the viral strains in Yeh and Leser were from 2014 and prior, we sought to evaluate the residues of interest in modern circulating populations by assessing mutational frequency over time. We found that VP3 I88 and VP1 E283 have been relatively fixed in viral populations since 2014; however, there has been more population-level diversity at VP1 positions 1, 2, and 98. As clinical AFM attributed to EV-D68 has declined since 2018, variation at these residues may contribute to changes in neurovirulence, although other factors such as host immunity, surveillance practices, and environmental influences are also likely to play a role.

## 2. Materials and Methods

### 2.1. Cells and Viruses

Human embryonic kidney (HEK) 293T cells (ATCC CRL-3216) and human RD cells (ATCC CCL-136) were obtained from ATCC (Manassas, VA, USA). Human induced pluripotent stem cells (iPSC, SCTi003-A) were obtained from STEMCELL Technologies (Seattle, WA, USA). HEK 293T and RD cells were maintained in Dulbecco’s Modified Eagle’s Medium (DMEM, Corning (Pittston, PA, USA)) supplemented with 10% fetal bovine serum (FBS, Biowest (Bradenton, FL, USA)) and 1% penicillin/streptomycin (Gibco (Miami, FL, USA)). Human iPSCs were maintained in mTeSR^TM^ Plus medium (STEMCELL Technologies) or Essential 8 medium (Thermofisher (Pittsburgh, PA, USA)) in flasks coated with 150 µg/mL Cultrex (R&D Systems (Minneapolis, MN, USA)). Cells were maintained in sterile cell culture incubators at 37 °C and 5% CO_2_ unless otherwise stated.

### 2.2. Cloning and Generation of rEV-D68

EV-D68 infectious clones were obtained through BEI Resources, National Institute of Allergy and Infectious Diseases (NIAID), National Institute of Health (rUSA/IL/2014-18952 from pUC19-EVD68_49131 (NR-52011) and rUSA/Fermon from pUC19-R-Fermon) (NR-52375). Point mutations were inserted into the pUC19-EVD68_49131 using the NEBuilder HiFi DNA Assembly Method based on previously published literature [6]. To generate the VP3 mutation, the codon for isoleucine at site 88 was replaced with a valine (I88V) (rVP3-I88V-MO) (Table 1). To replicate the mutations from Yeh et al. [3] in VP1, we altered three amino acids (rVP1-3AA-MO): leucine at site 1 to proline (L1P), valine at site 148 to alanine (V148A), and lysine at site 282 to arginine (K282R). Yeh et al. also identified a fourth point mutation at site 283 in VP1 (glycine to glutamic acid at site 283 (G283E)) [3]. However, our EV-D68 18952 strain already encodes E283, therefore we did not make a point mutation at VP1 site 283. To generate four amino acid mutations from Leser et al. [4] in VP1 (rVP1-4AA-CA), the following changes were made: leucine at site 1 to an isoleucine (L1I), asparagine at site 2 to aspartic acid (N2D), threonine at site 98 to an alanine (T98A), and glutamic acid at site 283 to a lysine (E283K). To generate rVP3-I88V/VP1-4AA-CA combined mutations, we made five-point mutations, one in VP3 (I88V) and four in VP1 (L1I/N2D/T98A/E283K). PCR primers were created according to Table 1.

### 2.3. Transfection and Virus Rescue

HEK293T cells were seeded into 100 mm^2^ dishes. Plasmids were transfected with pCAGGS T7 at a ratio of 10:1 with the Transit-LT1 transfection reagent (Mirus Bio (Madison, WI, USA)) into HEK293T cells. Cells were incubated at 33 °C and 5% CO_2_ until cytopathic effects were visible. Cells were scraped from the dish and collected with supernatant.

Viral stocks were obtained by incubation of transfected lysates on RD cells. RD cells were seeded with 20 million cells in 150 mm^2^ dishes. Half of the transfection lysate was added to RD cells and rocked at room temperature for one hour to adsorb the virus. Mock infected cells received cell culture media only. Inoculum was aspirated from cells and fresh cell growth media was added. Cells were incubated at 33 °C and 5% CO_2_ until cytopathic effects. Viral stocks were harvested and purified using sucrose cushion centrifugation, as previously described [7].

### 2.4. Growth Kinetics in Human Cell Lines

RD cells were seeded at 200,000 cells per well and grown in a 24-well tissue culture plate. RD cells were infected with rescued recombinant virus at an MOI of 0.01 plaque forming units (PFU)/cell. Cells were inoculated in biological triplicate with the indicated recombinant virus or mock-infected with PBS. Virus was adsorbed onto cells for one hour at room temperature with rocking. Following one hour, the inoculum was aspirated, cells were washed three times with PBS, and fresh growth medium was added to cells. RD cells were incubated for 96 h at 33 °C and 5% CO_2_. Supernatants were taken at 0, 24, 48, 72, and 96 hpi and stored at −80 °C. Infectious virus was quantified by 50% tissue culture infectious dose assay (TCID_50_) on RD cells in technical triplicate. TCID_50_ titers were enumerated by the Spearman–Kärber method.

### 2.5. Differentiation and Propagation of Human Spinal Cord Organoids

Three-dimensional spinal cord (3-DiSC) organoids were differentiated from iPSC cells as previously described [5]. iPSC cells were seeded at a density of 9000 cells per well into a 96-well round-bottom low-adhesion plates using Accumax (Sigma (Milwaukee, WI, USA)). Cells were plated in differentiation medium for 14 days supplemented with designated growth factors [5]. After 14 days, organoids were transferred to a tissue culture plate and cultured in suspension in N2B27 medium [DMEM/F-12 (Gibco), neurobasal medium (Gibco) (1:1), 0.5% (*vol*/*vol*) N2 supplement (ThermoFisher) and 1% (*vol*/*vol*) B27 supplement without vitamin A (Gibco)] supplemented with 1 mM L-glutamine (ThermoFisher), 0.1 mM β-mercaptoethanol, 0.5 µM ascorbic acid, 10 ng/mL brain-derived neurotrophic factor (BDNF, STEMCELL Technologies), 10 ng/mL glial cell line-derived neurotrophic factor (GDNF, STEMCELLL Technologies), and 100 nM retinoic acid (Tocris (Minneapolis, MN, USA)). All experiments in hSCOs were performed at 14 days post-differentiation and were allowed to mature up to 18 days post-differentiation, as previously described [5].

### 2.6. Growth Kinetics in Human Spinal Cord Organoids

Pools of 12 3-DiSC hSCOs were inoculated with rescued recombinant virus in biological triplicate at 14 days post-propagation. hSCOs were infected with 10^4^ PFU per pool in N2B27 medium. Pools were either infected with the indicated recombinant virus or with a mock inoculum of PBS. Virus was adsorbed on hSCOs for one hour at room temperature with rocking. After one hour, inoculum was aspirated, and organoids were washed three times with PBS. Pools of hSCOs were then transferred to new plastic to ensure there was no residual inoculum present and fresh growth medium was added. hSCOs were incubated for 96 h at 33 °C and 5% CO_2_. Supernatants were taken at 0, 24, 48, 72, and 96 hpi and stored at −80 °C. At 96 hpi, hSCOs were fixed with 4% paraformaldehyde in PBS for IF staining. Infectious virus was quantified by TCID_50_ on RD cells in technical triplicate. TCID_50_ titers were enumerated by the Spearman–Kärber method.

### 2.7. Immunofluorescence Staining

IF staining on fixed hSCOs was completed as previously described [5]. hSCOs were washed with 1% PBS-BSA and PBS-Tween at 4 °C. The remaining washes and antibody dilutions were diluted in organoid wash buffer (0.1% Triton X-100 (*v*/*v*), 0.2% BSA (*m*/*v*) in PBS). Primary antibodies VP1 (Genetex (Irvine, CA, USA), GTX132313), Phalloidin stain 543 (Biotium (Fremont, CA, USA), 00043-T), S100 beta (Thermofisher, 15146-1-AP), Tuj1 (Novus Biologicals (Littleton, CO, USA), NB100-1612), and double-stranded RNA (dsRNA) (Sigma, MABE1134-100UL) were used at a 1:250 dilution. Secondary antibodies Alexa Fluor^TM^ 488 (A-11001), Alexa Fluor^TM^ 647 (A21449), and Alexa Fluor^TM^ 546 (A-11035) were used at a 1:1000 dilution. DAPI (4′,6-diamidino-2-phenylindole, dihydrochloride) (ThermoFisher, D1306) was used at a 1:300 dilution. hSCOs were cleared for 30 min at with 60% glycerol in 2.5 M fructose before mounting and imaging.

### 2.8. Confocal Imaging

Microscope slides were imaged on a Leica Stellaris 5 confocal microscope. Volumetric Z-stacks were imaged with a 20× objective by oil immersion. Sequential scanning was used with 2× line averaging by frame. The following parameters were used for Figures 2 and 4: dsDNA was measured at 403 nm (4′,6-diamidino-2-phenylindole (DAPI)), VP1 was measured at 488 nm, and phalloidin stain was measured at 543 nm. For Figure 3: DAPI was measured at 403 nm, dsRNA was measured at 488 nm, S100β was measured at 546 nm, and Tuj1 at 647. Images were processed with FIJI 2 version 2.9.0.

### 2.9. Statistical Analysis

All statistical analyses were performed using Graphpad Prism version 10. The statistical test used for each figure is indicated in the figure legend. All experiments were performed in at least biological triplicate corresponding to each experimental condition. Each biological replicate consisted of an average of three technical replicates.

### 2.10. Analysis of Substitution Frequencies

EV-D68 FASTA sequences (*n* = 7468) were downloaded from the Bacterial and Viral Bioinformatics Resource Center (BV-BRC, accessed 26 November 2025). Partial sequences or sequences isolated outside of the 2014–2024 timespan were excluded, leaving 1968 for analysis. Sequences were aligned in NextClade v3.21.2 (https://clades.nextstrain.org) [8] using the EV-D68 dataset with reference Fermon (accession no. AY426531.1) [9] as a basis for QC, clade assignment, and mutation calling. Sequences that failed QC were excluded (*n* = 2). Data were visualized in R (version 4.4.1) using the expss (version 0.11.7) and areaplot (version 2.1.3) packages.

## 3. Results

### 3.1. Recombinant EV-D68 with Mutations in VP1 and VP3 Replicate Efficiently in RD Cells

**Figure 1:**
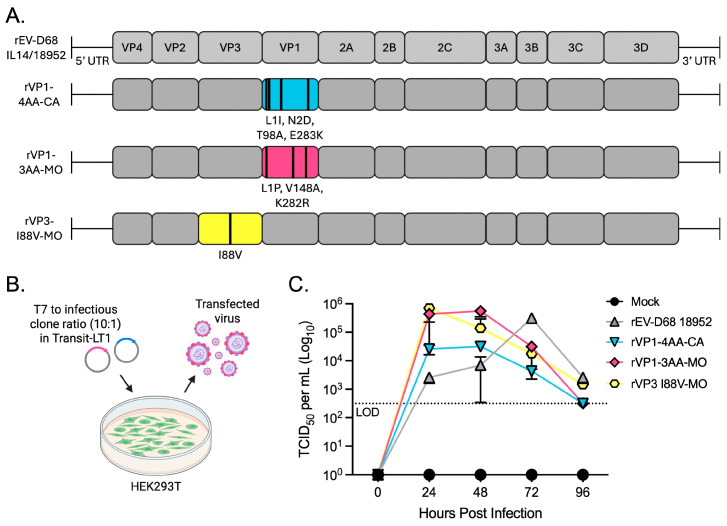
Confirmation of recombinant EV-D68 with VP1 and VP3 mutations. (**A**) Schema of proposed recombinant viruses with point mutations in either VP1 or VP3. Gray represents rEV-D68 18952; blue—rVP1-4AA-CA (L1I/N2D/T98A/E283K); pink—rVP1-3AA-MO (L1P/V148A/K282R); and yellow—rVP3-I88V-MO. (**B**) Infectious clones were cloned into pUC19-EV-D68_49131 and were transfected with pCAGGS Y7 at a ratio of 10:1 with the Transit-LT1 transfection reagent (Mirus Bio) into HEK293T cells. Cells were incubated until cytopathic effects were visible and then were collected. Viral stocks were obtained by incubation of transfected lysates on RD cells. Created with Biorender.com. Schematic is modeled from Jones et al. 2026 [6]. (**C**) RD cells were infected at an MOI 0.01 PFU/cell in biological triplicate with each recombinant virus. Viral titers were determined by TCID_50_ in RD cells. Black circle—mock; gray triangle—rEV-D68 18952; blue upside-down triangle—rVP1-4AA-CA; pink diamond—rVP1-3AA-MO; yellow hexagon—rVP3-I88V-MO.

We recreated three rEV-D68 viruses as previously described in the EV-D68 18952 backbone: one rEV-D68 with four amino acid changes in VP1 (designated rVP1-4AA-CA), one rEV-D68 with three amino acid changes in VP1 (designated rVP1-3AA-MO), and one rEV-D68 with a point mutation in VP3 (designated rVP3-I88V-MO) (Figure 1A) [3,4]. We then inserted point mutation fragments into the pUC19-EVD68_49131 vector using the NEBuilder HiFi DNA Assembly Method [6]. As controls, we also created plasmids expressing EV-D68 US/IL/14-18952 (designated rEV-D68 18952) and EV-D68 Fermon (designated rFermon) [5]. We then transfected plasmids into HEK293T cells with pCAGGS T7 at a ratio of 10:1 with the Transit-LT1 transfection reagent (Figure 1B). After observable cytopathic effects (CPE), we collected supernatants and created viral stocks.

To determine the replication competence of recovered rEV-D68s, RD cells were infected with each rEV-D68 at an MOI 0.01 PFU/cell. Supernatants were collected between 0 and 96 hpi at 24 h intervals followed by titer determination by TCID_50_. We found that infectious virus was detectable at 24 hpi for all recombinant viruses (Figure 1C). These data indicate that we successfully rescued replication-competent recombinant EV-D68 without growth defects in RD cells.

### 3.2. rVP3-I88V-MO and rVP1-4AA-CA Have Decreased Viral Replication in hSCOs

**Figure 2:**
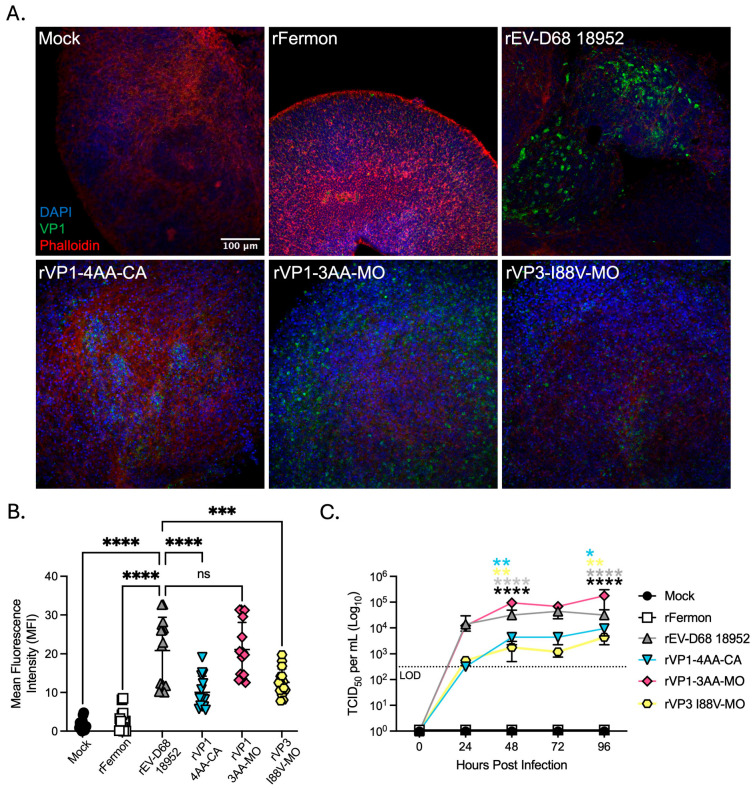
rVP1-4AA-CA and rVP3-I88V-MO result in decreased infection of hSCOs. (**A**) IF images of hSCOs infected with recombinant viruses. hSCOs were infected with 10^4^ PFU and were fixed in 4% paraformaldehyde at 96 hpi. hSCOs were then stained with DAPI (blue), VP1 (green), and phalloidin (red). (**B**) MFI of VP1 staining was quantified across five biological replicate images of each recombinant virus across multiple Z stacks and organoids. Quantified values were calculated using Fiji ImageJ version 2.16.0/1.5p. A one-way ANOVA with multiple comparisons to EV-D68 18952 was performed. (**C**) hSCOs were infected with 10^4^ PFU of each recombinant virus in biological triplicate. Supernatants were collected every 24 h up to 96 hpi. Viral titers were determined by TCID_50_ in RD cells. A two-way ANOVA with multiple comparisons to EV-D68 18952 was performed. Black asterisks—compared to mock; gray asterisks—compared to rFermon; blue asterisks—compared to rVP1-4AA-CA; and yellow asterisks—compared to rVP3-I88V-MO. Black circle—mock; white square—rFermon; gray triangle—rEV-D68 18952; blue upside-down triangle—rVP1-4AA-CA (L1I/N2D/T98A/E283K); pink diamond—rVP1-3AA-MO (L1P/V148A/K282R); and yellow hexagon—rVP3-I88V-MO. ns—no significance, * *p* < 0.05, ** *p* < 0.005, *** *p* < 0.0005 **** *p* < 0.00005.

We next wanted to determine if point mutations VP1 or VP3 influence neurotropism in our hSCO model. hSCOs were mock-infected or infected with 10^4^ PFU rEV-D68. We collected infected hSCOs at 96 hpi and stained hSCOs for VP1, an EV-D68 structural protein, and phalloidin, a filamentous actin stain (Figure 2A). We then quantified the mean fluorescence intensity (MFI) of VP1 staining across all hSCOs to assess both the frequency and brightness of VP1 per cell (Figure 2B). We found that there was little VP1 staining in mock- or rFermon-infected hSCOs, consistent with previously published results using the clinical strain [5]. We also found that compared to rEV-D68 18952, there was a significant decrease in VP1 staining in rVP3-I88V-MO and rVP1-4AA-CA.

As a complementary approach, we then assessed rEV-D68 growth in hSCOs. hSCOs were pooled as described previously and organoids were infected with 10^4^ PFU per pool (Figure 2C). We found that rFermon did not produce a measurable titer in hSCOs consistent with previously published data using the clinical strain [5]. At 48 and 96 hpi, rVP3-I88V-MO and rVP1-4AA-CA resulted in decreased viral titer compared to the control strain rEV-D68 18952. These data suggest that rVP3-I88V-MO and rVP1-4AA-CA, but not rVP1-3AA-MO, have decreased fitness in cells of the human CNS.

### 3.3. rVP1-3AA-MO Colocalizes Primarily with Glial Cells Whereas rVP1-4AA-CA and rVP3-I88V-MO Colocalize Primarily with Neuronal Cells

**Figure 3:**
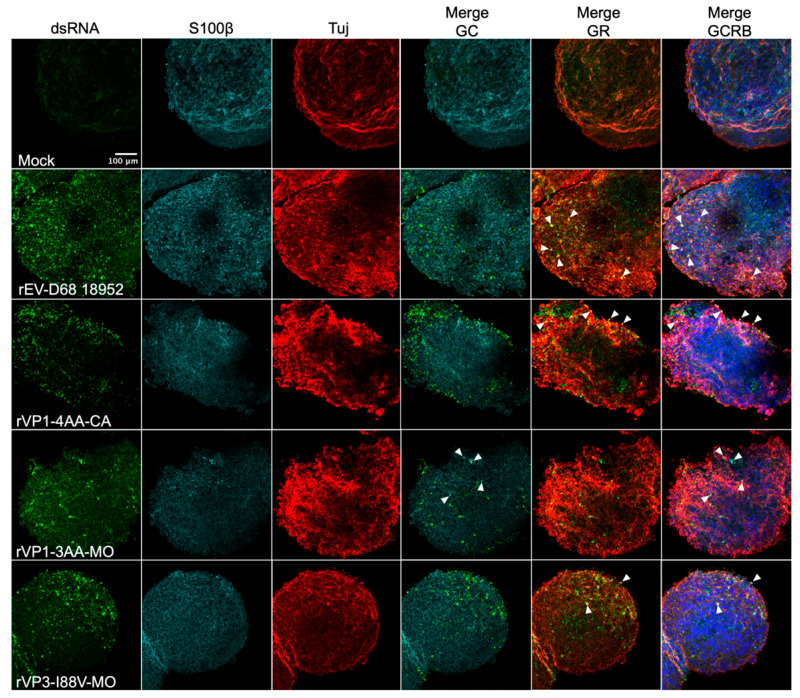
rVP1-3AA-MO colocalizes primarily with glial cells, whereas rVP1-4AA-CA and rVP3-I88V-MO colocalize primarily with neuronal cells. hSCOs were infected at 10^4^ PFU of each recombinant virus. hSCOs were then fixed in 4% paraformaldehyde at 96 hpi. hSCOs were then stained with DAPI (blue), dsRNA (green), S100β (cyan), and Tuj (red). White arrows indicate colocalization of either dsRNA and S100B or dsRNA and Tuj. Scale is 100 µM.

We have previously characterized the diversity and abundance of cell types in hSCOs using single-cell RNA sequencing (scRNAseq) [10]. hSCOs are composed of neuronal and glial cells, including astrocytes, oligodendrocytes, and their progenitors. We sought to determine if VP1 and/or VP3 point mutations alter which cell types are infected in hSCOs. hSCOs were mock-infected or infected with 10^4^ PFU rEV-D68 for 96 h and stained with double-stranded RNA (dsRNA); S100 beta (S100β), a glial cell marker; and Tuj1, a neuronal cell marker (Figure 3). We found that during rEV-D68 18952 infection, dsRNA and Tuj colocalize, suggesting neuronal infection. We found similar cellular tropism when hSCOs were infected with rVP1-4AA-CA and rVP3-I88V-MO. Interestingly, we found that rVP1-3AA-CA primarily colocalized with glial cells (dsRNA and S100β).

### 3.4. rVP3-I88V/VP1-4AA-CA Results in Decreased Viral Replication in hSCOs Compared to rEV-D68 18952

**Figure 4:**
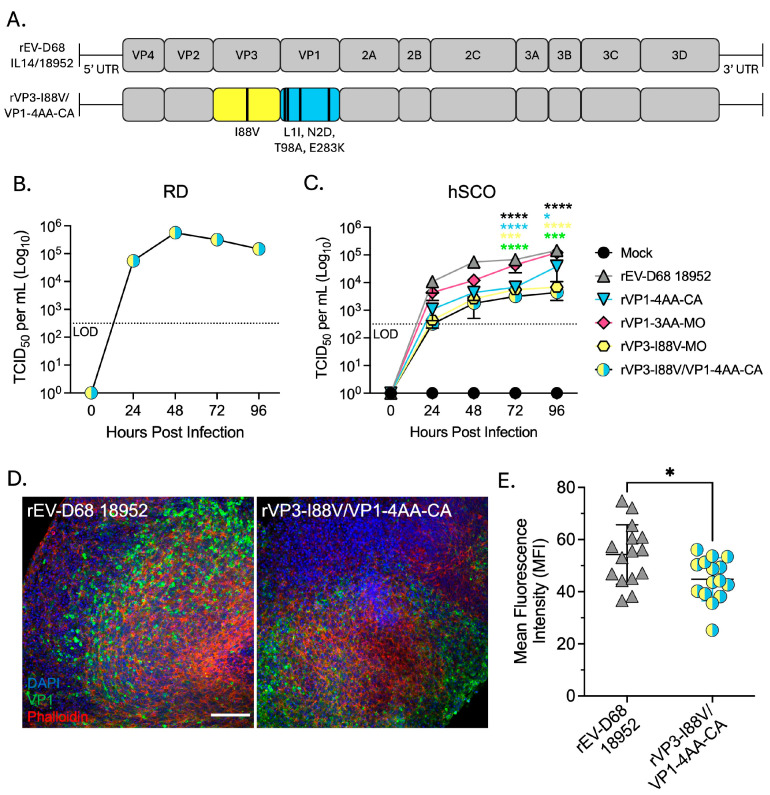
Combining VP1-4AA-CA and VP3-I88V-MO point mutations results in decreased viral titers compared to rEV-D68 18952. (**A**) Schema of rVP3-I88V/VP1-4AA-CA recombinant viruses with VP3 I88V and VP1 L1I/N2D/T98A/E283K. Gray represents rEV-D68 18952, and yellow and blue represent rVP3-I88V/VP1-4AA-CA. (**B**) RD cells were infected at an MOI 0.01 PFU/cell in biological triplicate with rVP3-I88V/VP1-4AA-CA. Viral titers were determined by TCID_50_ in RD cells. (**C**) hSCOs were infected with 10^4^ PFU of either recombinant viruses in biological triplicate. Supernatants were collected every 24 h up to 96 hpi. Viral titers were determined by TCID50 in RD cells. A two-way ANOVA with multiple comparisons to EV-D68 18952 was performed. Black asterisks—compared to mock; blue asterisks—compared to rVP1-4AA-CA; yellow asterisks—compared to rVP3-I88V-MO; green astericks—compared to rVP3-I88V/VP1-4AA-CA. Black circle—mock; gray triangle—rEV-D68 18952; blue upside-down triangle—rVP1-4AA-CA (L1I/N2D/T98A/E283K); pink diamond—rVP1-3AA-MO (L1P/V148A/K282R); yellow hexagon—rVP3-I88V-MO; yellow and blue circle—rVP3-I88V/VP1-4AA-CA. (**D**) IF images of hSCOs infected with rEV-D68 18952 or rVP3-I88V/VP1-4AA-CA. hSCOs were infected with 10^4^ PFU and were fixed in 4% paraformaldehyde at 96 hpi. hSCOs were then stained with DAPI (blue), VP1 (green), and phalloidin (red). Scale bar is 100 µm. (**E**) MFI of VP1 staining was quantified across five biological replicate images of each recombinant virus across multiple Z stacks and organoids. Quantified values were calculated using Fiji ImageJ. An unpaired student *t*-test was performed (* *p* < 0.05, *** *p* < 0.0005, **** *p* < 0.00005).

After determining that both rVP3-I88V-MO and rVP1-4AA-CA resulted in decreased viral titers and VP1 expressions, we wanted to determine if combining all five mutations into a single recombinant virus would have an additive effect on neurotropism. We inserted VP3 I88V and VP1 L1I, N2D, T98A, and E283K point-mutation fragments (designated rVP3-I88V/VP1-4AA-CA) into the pUC19-EVD68_49131 vector (Figure 4A), as previously described, followed by transfection into HEK293T cells [6]. We successfully recovered this rEV-D68 and rVP3-I88V/VP1-4AA-CA which replicated efficiently in RD cells between 0 and 96 hpi (Figure 4B).

We then infected hSCOs at 14 days post-differentiation with 10^4^ PFU of rVP3-I88V/VP1-4AA-CA and rEV-D68 18952 and assessed viral titer and VP1 expression and localization with IF at 96 hpi (Figure 4C–E). Compared to rEV-D68 18952, we found that rVP3-I88V/VP1-4AA-CA viral titer and VP1 expression was significantly reduced at each evaluated time point until 96 hpi. We also we created rEV-D68 with each single amino acid mutation from rVP1-4AA-CA (Supplemental Table S1). We found that there was no significant difference in viral titer in hSCOs when infected with recombinant viruses compared to rEV_D68 18952 (Supplemental Figure S1).

### 3.5. Substitution Frequency of EV-D68 VP1 and VP3 Residues of Interest Since 2014

**Figure 5:**
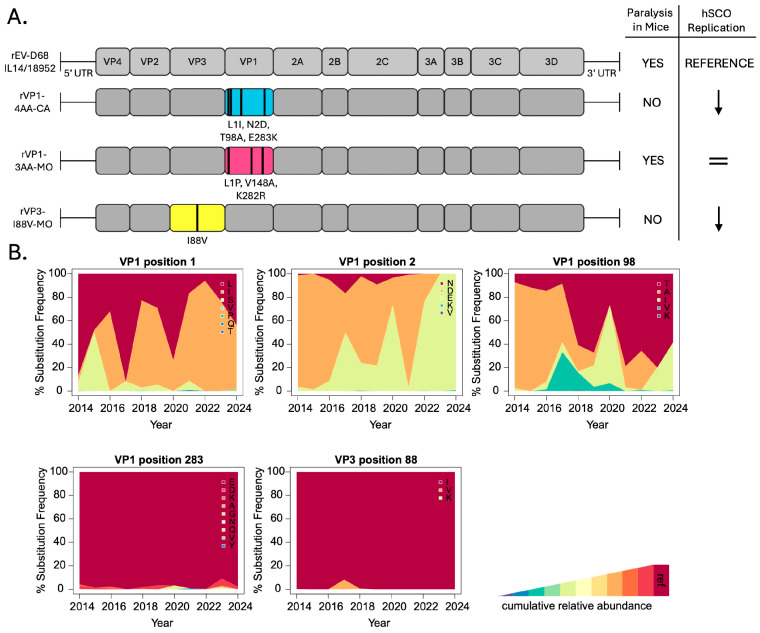

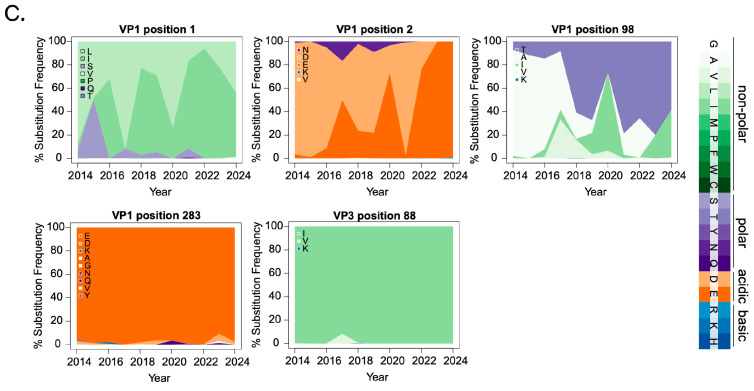
Frequency of VP1 and VP3 point mutations in clinical isolates that have circulated between 2014 and 2024. (**A**) Schema of VP1 and VP3 recombinant viruses and the effects they have on mice paralysis and hSCO replication. Gray represents rEV-D68 18952; blue—rVP1-4AA-CA (L1I/N2D/T98A/E283K); pink—rVP1-3AA-MO (L1P/V148A/K282R); and yellow—rVP3-I88V-MO. Black arrow—decreased viral replication in hSCO compared to reference strain (EV-D68 18952); black ‘=’—similar rate of viral replication to EV-D68 18952. (**B**) Global EV-D68 sequences from 2014 to 2024 were obtained from the Bacterial and Viral Bioinformatics Resource Center (*n* = 1968) and aligned using NextClade. Amino acid substitutions were visualized in an area plot as a percentage of the total sequences analyzed in each year. The identity of each residue in the reference strain, EV-D68 18952, is shown in red, and substitutions are colored according to relative abundance in each year. (**C**) Amino acid types were overlaid onto the substitution frequencies shown in panel (**B**).

In 2014, the CDC began tracking AFM cases. EV-D68 circulated biannually until 2020, when EV-D68 circulation was disrupted due to non-pharmaceutical interventions for COVID-19 [11,12]. While EV-D68 returned robustly in 2022, there was not a corresponding spike in AFM cases [13,14]. Therefore, we sought to analyze available sequencing data to assess if frequencies of these neurotropism-driving residues of VP3 and VP1 at the population level provided a potential explanation for the altered clinical neurotropism phenotype seen since 2022. We assessed 1968 sequences from strains isolated between 2014 and 2024 worldwide. We found that since 2014, VP3 I88, the virulent residue, has been fixed at the population level with only three clinical isolates (2016–2018), with VP3 V88 (Figure 5A,B). As for VP1, we found that E283 is very stable, with only seven occurrences of K283 since 2014. The other VP1 sites of interest were more variable, however, with I1 occurring in 61% of sequences, D2 occurring in 59%, and A98 occurring in 43%, potentially explaining the changing clinical manifestations. Some substitutions resulted in dramatic shifts in amino acid side-chain properties as well, with VP1 T98 shifting from polar to non-polar (Figure 5C).

## 4. Discussion

While several viruses have been associated with recent outbreaks of AFM, EV-D68 is among the most frequently detected [15]. Although EV-D68-associated AFM is generally thought to result from viral replication in the spinal cord, direct detection of the virus at the site of neurological injury is often limited, complicating definitive attribution [16,17,18,19]. Previous research in immunocompromised murine models has demonstrated that EV-D68 containing point mutations in structural proteins VP3 and VP1 resulted in decreased paralysis and less morbidity [3,4]. We sought to investigate if VP3 and VP1 directed neurotropism or cellular tropism in a human CNS model, if those attenuating mutations were additive, and if these mutations persisted in modern EV-D68 clinical sequences, perhaps accounting for the decrease in AFM associated with EV-D68 since 2022.

Each of these prior studies used different inoculation routes, with Yeh et al. using both IC and IP injection and Leser et al. using intramuscular (IM) injection [3,4]. Viruses that are injected by IM and IP enter the CNS indirectly by moving into nearby blood vessels to enter the blood stream [20,21]. Viruses injected by IM or IP then must cross the blood–brain barrier (BBB) or exploit retrograde axonal transport to move from the peripheral nerves directly to motor neurons within the spinal cord [20,21]. IC injection, however, delivers viral infection directly to the CNS, bypassing the BBB. Similarly, infection of hSCOs mirrors direct inoculation of the CNS, allowing for the study of tropism. Leser et al. did not assess IC infection or infection of neurons in vitro [4], so it is not known if the infection defect is due to an issue in cellular tropism or a defect of trafficking to and accessing the CNS. They also did not assess each mutation individually; therefore, it is unknown if a combination of mutations is required to modify EV-D68 neurovirulence.

The cellular composition of hSCOs, characterized by gene and protein expression, includes neurons, astrocytes, oligodendrocytes, and progenitor glial populations [5,10]. We discovered that rVP1-3AA-MO has altered cellular tropism for glial cells as compared to parental rEV-D68 18952 which infects neurons (Figure 3). Cellular tropism of EV-D68 is likely defined by multiple entry factors, including the recently identified major facilitator superfamily domain-containing 6 (MFSD6) [22,23]. MFSD6 mediates EV-D68 attachment and entry in immortalized cell lines, primary respiratory cells, and primary astrocytes [22,23]. Interestingly, rVP1-3AA-MO includes a point mutation in V148A, the amino acid directly next to G149, which interacts with MFSD6 [3,23]. Perhaps this point mutation has allowed a change in shape or charge of VP1, causing the virus to modify receptor or cofactor interactions in a cell-type-dependent manner.

In 2014, the CDC began tracking AFM cases; however, in 2022, AFM cases did not increase concordant with EV-D68 circulation [13,14]. The reasons for this decoupling are unknown. Yeh et al. determined the prevalence of VP3-I88V in clinical isolates from the former Virus Pathogen Resource (ViPR) from 2014 [3]. Consistently, their analysis found that only two out of 676 collected sequences had V88 present in VP3. Our analysis of an additional 1292 sequences from the recently consolidated Bacterial and Viral Bioinformatics Resource Center (BV-BRC) found only one more virus with V88. Past and present circulating clinical isolates of EV-D68 have VP3 I88 as the prominent amino acid (Figure 5B). We therefore anticipate that it is unlikely that I88V is the main driver of decreased neurovirulence in recent clinical EV-D68 strains.

Another comprehensive examination of EV-D68 sequences in 2023 (published in 2025) investigated European clinical samples and compared them to Leser et al.’s findings [4,24]. They found that A98T was found in all European sequences while E283K was identified in all except two. Finally, they identified that the VP1 N2D substitution has continued to evolve into D2E. In 2022, the Johns Hopkins Health System also identified a major shift to VP1 E2 which has remained the primary circulating residue in isolates from Baltimore, MD [25]. Similarly, we found that compared to reference strain EV-D68 18952, there are only seven occurrences of K283 since 2014, indicating that E283 is prominent not only in European isolates but globally. We anticipate that VP1 site 283 is not a primary driver of neuropathogenesis in recent circulating strains due to the limited variability in amino acids and E283 continuing to be the major variant (Figure 5B,C). By contrast, VP1 sites 1, 2, and 98 have a greater percent substitution frequency between 2014 and 2024. I1 occurred in 61% of sequences, and A98 occurred in 43% (Figure 5B). Like Hirvoven et al., we found that D2 occurred in 59% of isolates and later shifted to E (39% relative abundance). These data indicate that there is selective pressure on VP1 at these specific sites. VP1 site 2 has evolved from a basic amino acid (N) to primarily acidic amino acids (D and E). Interestingly, D and E are biochemically similar, differing only by an extra methylene in the glutamic acid side-chain. VP1 site 98 similarly has evolved from a polar amino acid (T) to different types of non-polar amino acids (A, I, and V). Perhaps changes in amino acid charge and solubility have altered enzymatic active sites, substrate and receptor bindings, and protein–protein interactions, rendering recent strains to be non-neuropathogenic.

Additionally, both VP3 and VP1 are structural proteins that form the viral capsid with VP2 while VP4 is nestled within the particle to stabilize the structure. VP1 also functions by mediating viral receptor binding, host immune evasion, and regulation of tissue tropism, and encodes epitopes that provide the basis for EV-D68 serotyping [26,27,28]. Structurally, VP1 also has two surface-exposed loops, the BC-loop and DE-loop, both of which interact with host cell receptors and can modulate receptor binding affinity [27]. For example, host receptor MFSD6 interactions are driven by the flexibility of the BC-loop [22]. Because the BC-loop is composed of amino acids between positions 90–103 of VP1 [29], there is a possibility that VP1 site 98 helps drive host cell receptor interactions and may account for decreased AFM in the 2022 and 2024 seasons.

## 5. Conclusions

In this study, we evaluated previously described EV-D68 neurovirulence determinants in a multicellular human CNS model. Infection of hSCOs with recombinant viruses resulted in similar replication results as previously published murine models, further demonstrating model complementarity with the new discovery that rVP1-3AA-MO altered cellular tropism in our human model. We found that combining VP1 L1I/N2D/T98A/E283K and VP3 I88V mutations did not result in an additive or abortive phenotype in hSCOs. We then evaluated the frequency of these point mutations in reported clinical EV-D68 isolates since 2014, and discovered significant ongoing evolution at three sites, VP1 1, 2, and 98, perhaps explaining changes in clinical phenotype and lessened neurovirulence. These data show that EV-D68 continues to rapidly adapt and evolve, thus emphasizing the importance for continued EV-D68 surveillance and study of contemporary strains. Comprehensive and systematic evaluation of neurovirulence drivers of other structural and non-structural proteins will provide additional insight into the clinical manifestations of this continually evolving pathogen.

## Figures and Tables

**Table 1:** Primers used for NEBuilder HiFi DNA assembly to generate VP3 and VP1 recombinant EV-D68 (rEV-D68).

rEV-D68	Fragment Number	Forward Primer (5′ to 3′)	Reverse Primer (5′ to 3′)
rVP1-4AA-CA	1	GACAAATAGACCACTTACATGCAGCAGAGGC	GTTCTTGTCTGCTTGTGCCGCAGACG
	2	TCTGCGGCACAAGCAGACAAGAACT	AGCATTAAGCGCATTTGGTGCTCTCTTTTTAC
	3	GGTAAAAAGAGAGCACCAAATGCGCTTAATGC	CTCTCCATTGTGCCATGCGAGATAGCACA
	4	GCTATCTCGCATGGCACAATGGAGAGGAG	TGCATGTAAGTGGTCTATTTGTCCAATGTCAGGGC
rVP1-3AA-MO	1	CTGACATTGGACAACCCAACCACTTACATGC	CAGGAAGACCGGCGTATGTGTTGTTACTACTACC
	2	AGTAACAACACATACGCCGGTCTTCCTGAC	TAAGCGCATTTGGTGCTCTCTCTCTACCTTTAT
	3	AGGTAGAGAGAGAGCACCAAATGCGC	CTCTCCATTGTGCCATGCGAGATAGCACA
	4	GCTATCTCGCATGGCACAATGGAGAGGAG	GTAAGTGGTTGGGTTGTCCAATGTCAGGGCTG
rVP3-I88V-MO	1	ACATTCCACTGGACGTGCAGTTGGATGGG	CTCTCCATTGTGCCATGCGAGATAGCACA
	2	GCTATCTCGCATGGCACAATGGAGAGGAG	CATCCAACTGCACGTCCAGTGGAATGTTA
rVP3-I88V/VP1-4AA-CA	1	ACATTCCACTGGACGTGCAGTTGGATGGG	TGCATGTAAGTGGTCTATTTGTCCAATGTCAGGGC
	2	GACAAATAGACCACTTACATGCAGCAGAGGC	GTTCTTGTCTGCTTGTGCCGCAGACG
	3	TCTGCGGCACAAGCAGACAAGAACT	AGCATTAAGCGCATTTGGTGCTCTCTTTTTAC
	4	GGTAAAAAGAGAGCACCAAATGCGCTTAATGC	CTCTCCATTGTGCCATGCGAGATAGCACA
	5	GCTATCTCGCATGGCACAATGGAGAGGAG	CATCCAACTGCACGTCCAGTGGAATGTTA

## Data Availability

The original contributions presented in this study are included in the article/Supplementary Material. Further inquiries can be directed to the corresponding author.

## Supplementary Materials

The following supporting information can be downloaded at: https://www.mdpi.com/article/10.3390/v18060619/s1. Supplemental Table S1. Primers used for Q5^®^ Site-Directed Mutagenesis Kit to generate VP1 rEV-D68 single amino acid mutations. Supplemental Figure S1. Single point mutations in VP1 do not result in a significant difference in viral titer compared to rEV-D68 18952 in hSCOs.

## Abbreviations

The following abbreviations are used in this manuscript:

EV-D68	Enterovirus D68
AFM	Acute flaccid myelitis
CNS	Central nervous system
hSCO	Human spinal cord organoid
IP	Intraperitoneal
IC	Intracranial
IM	Intramuscular
TCID_50_	50% tissue culture infectious dose assay
PFU	Plaque forming units
