# Supplementary material for: Enterovirus D68 VP1 and VP3 Determine Neurotropism in Human Spinal Cord Organoids
Source: Viruses. 2026 May 28;18(6):619. doi: 10.3390/v18060619 (PMC13307885; doi:10.3390/v18060619)
Supplement: Supplementary file 1 [file viruses-18-00619-s001.zip › Supplementary Materials FINAL.pdf]

Supplementary Materials:

Supplemental Table S1. Primers used for Q5® Site-Directed Mutagenesis Kit to generate VP1 rEV-D68 single amino acid mutations.

**rEV-D68**	**Forward Primer (5′ to 3′)**	**Reverse Primer (5′ to 3′)**
rVP1-L1I	GACAAATAAACCACTTACATGCAGCAGAGGC	CAATGTCAGGGCTGTCTCTCATTAATCTGAGG
rVP1-N2D	ATTGGACAATTAGACCACTTACATGCAGCAGAG	GTCAGGGCTGTCTCTCATTAATCTGAGGGA
rVP1-T98A	TCTGCGGCACAAGCAGACAAGAACT	CGAAGTATGATCTTTGTATTCAAAACTTCTCTTTGATACTAAAGCTG
rVP1-E283K	GGTAAAAAGAGAGCACCAAATGCGCTTAATGC	TTTATAATTTGCATTTGCAATGCTCATGTATGGGAGAGT

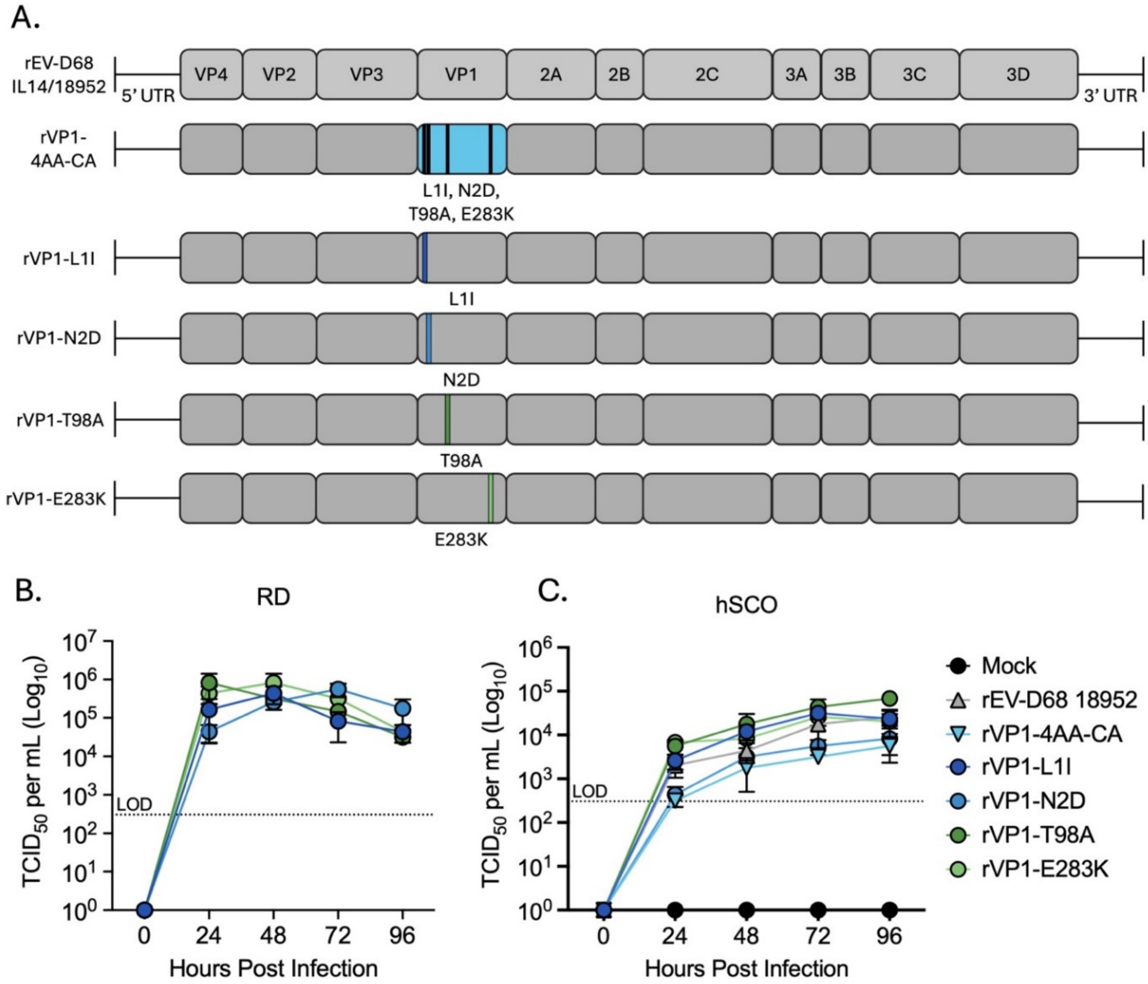

s

Supplemental Figure S1. Single point mutations in VP1 do not result in a significant difference in viral titer compared to rEV-D68 18952 in hSCOs. A.) Schema of rVP1-L1I, rVP1-N2D, rVP1-T98A, and rVP1-E283K recombinant viruses. Gray represents rEV-D68 18952, light blue represents rVP1-4AA-CA, dark blue rVP1-L1I, medium blue represents rVP1-N2D, dark green represents rVP1-T98A, and light green represents rVP1-E283K. B.) RD cells were infected at an MOI 0.01 PFU/cell in biological triplicate with each recombinant virus. Viral titers were determined by $TCID_{50}$ in RD cells. C.) SCTi003A hSCO were infected with 10^4 PFU of each recombinant virus in biological triplicate. Supernatants were collected every 24 hours up to 96 hpi. Viral titers were determined by $TCID_{50}$ in RD cells. A two-way ANOVA with multiple comparisons to EV-D68 18952 was performed (all multiple comparisons to rEV-D68 18952 were not significant). Black circle – mock, gray triangle - rEV-D68 18952, light blue upside-down triangle – rVP1-4AA-CA (L1I/N2D/T98A/E283K), dark blue circle – rVP1-L1I, blue circle rVP1-N2D. dark green circle – rVP1-T98A, and light green circle – rVP1-E283K.
